# Contribution of the Gut Microbiome to the Perpetuation of Inflammation in Crohn's Disease: A Systematic Review

**DOI:** 10.7759/cureus.67672

**Published:** 2024-08-24

**Authors:** Sai Pavitra Paidimarri, Shriya Ayuthu, Yashkumar D Chauhan, Parikshit Bittla, Amna A Mirza, Moyal Z Saad, Safeera Khan

**Affiliations:** 1 Internal Medicine, California Institute of Behavioral Neurosciences & Psychology, Fairfield, USA; 2 Medicine, California Institute of Behavioral Neurosciences & Psychology, Fairfield, USA

**Keywords:** disease progression, dysbiosis, inflammation, gut microbiome, crohn’s disease

## Abstract

Crohn's disease (CD) is a sub-type of inflammatory bowel disease (IBD) with a characteristic relapsing and remitting inflammation involving the gastrointestinal (GI) tract. Although there are several medications to relieve the symptoms, there is no definite cure for the condition. This paper highlights how CD affects our gut flora, which subsequently leads to the perpetuation of inflammation. This review was conducted according to Preferred Reporting Items for Systematic Review and Meta-Analysis (PRISMA) 2020 guidelines using PubMed, ScienceDirect, Multidisciplinary Digital Publishing Institute (MDPI), and Google Scholar as sources for relevant literature. After applying the quality appraisal tools, we finalized 11 articles for the paper. Inflammation seen in CD leads to dysbiosis, where there is a reduction in beneficial microbes such as *Faecalibacterium* and *Roseburia* species and an increase in pathogenic microbes such as *Escherichia* and *Proteus* species. This difference in gut microbes disrupts barrier function and immune processes in the intestine, contributing to the worsening of inflammation seen in CD. Several studies have been carried out to understand this complex relationship between the gut microbiome (GM) and CD, as it may serve as a potential novel therapeutic alternative, necessary as CD's burden is increasing globally.

## Introduction and background

The incidence and prevalence of Crohn's disease (CD) are increasing worldwide; for example, the prevalence in Canada in 2014 was 321 cases per 100,000, which increased to 410 cases per 100,000 in 2023 [1]. According to the Centers for Disease Control and Prevention (CDC), the number of Americans with CD was approximately 5,65,000 in 2013, and hospitalizations increased from 1,20,000 in 2003 to 1,96,000 in 2013 [2]. CD is one of the subtypes of inflammatory bowel disease (IBD), which involves transmural inflammation affecting the entire gastrointestinal (GI) tract [3]. It is a chronic relapsing and remitting condition with a wide range of symptom presentations, such as fatigue and diarrhea (it can be watery or bloody depending on the involved area), weight loss, and abdominal pain [4]. Although there are several proposed etiopathology involved in the disease, a few well-studied ones include: (1) environmental factors such as diet, lifestyle, and smoking, which are well-established; (2) genetic, many pathways are being investigated, among which the nucleotide-binding oligomerization domain-containing protein 2 (NOD-2) dependent signaling pathway and autophagy-related protein 16-like 1 (ATG16L1) are mainly studied; and (3) microbial, especially the gut microbiome (GM), which is believed to play a role in immunological processes [5]. Currently, there is no cure for CD. Still, a few agents that help achieve remission are corticosteroids, 5-aminosalicylates, immunomodulators such as methotrexate, azathioprine, and mercaptopurine, and biologic agents such as infliximab and adalimumab [6]. Complications, such as strictures, abscesses, and fistulae formation, may often require surgery [7].

The microbiome is a realm of a mixture of bacteria, viruses, fungi, and archaea, a part of the human body that is necessary for various functions, including maintaining gut-mucosal barrier integrity [8]. GM is believed to play a vital role in several GI tract, cardiovascular, neurological, and other diseases, including CD [9,10]. The inflammation seen in CD reduces the protective bacteria, such as *Firmicutes* and *Bacteroidetes*, which are 90% of the microbiome, and increases potentially harmful bacteria, such as *E. coli* and *Proteobacteria* [11]. This imbalance in the GM, also known as gut dysbiosis (GD), disrupts immune homeostasis, thereby perpetuating the inflammation seen in CD [12]. GD also leads to increased intestinal permeability, which can be a reason for the chronic inflammation seen in CD [13].

Current research is mainly focused on various techniques to detect microbial sequencing, which helps understand the role of microbes in human health and immunity [14-16]. These studies also give us more knowledge on dysbiosis and the pathways involved in the disease process, especially its role in CD recurrence and severity, thereby identifying potential targets for treatment and prevention [17,18]. Although there are several theories on etiopathophysiology, there are no studies on the exact etiology or cure for CD [19]. Additionally, it is still unclear if dysbiosis is the causal or consequential factor leading to inflammation and how the disease affects microbial heterogeneity [13,20].

In this systematic review, we will discuss various pathways of GM and their metabolic products that prolong the inflammation seen in CD, which will help us understand the role of dysbiosis in the pathogenesis of CD.

## Review

Methods

This systematic review was conducted using the Preferred Reporting Items for Systematic Review and Meta-Analysis (PRISMA) 2020 guidelines [21].

Search Sources and Strategy

We used PubMed, ScienceDirect, Multidisciplinary Digital Publishing Institute (MDPI), and Google Scholar to search for relevant literature. We used a variety of combinations of Boolean words, including Crohn's disease, GM, and inflammation, to search all these databases. A search strategy: (“Crohn's Disease/microbiology"(Majr) OR “Crohn's Disease/physiopathology"(Majr)). AND. (“Gastrointestinal Microbiome/immunology"(Majr) OR “Gastrointestinal Microbiome/physiology"(Majr)) was developed using PubMed's MeSH database. Table 1 below shows all the databases used and the number of articles identified from each database.

**Table 1:** Search strategy MDPI: Multidisciplinary Digital Publishing Institute

Search Strategy/Keywords	Database	Number of Articles
(“Crohn Disease/microbiology"(Majr) OR “Crohn Disease/physiopathology"(Majr)). AND. (“Gastrointestinal Microbiome/immunology"(Majr) OR “Gastrointestinal Microbiome/physiology"(Majr))	MeSH-PubMed	80
Crohn’s disease.AND.Gut Microbiome	PubMed	1365
((Crohn's disease (Text Word)) AND (gut microbiome (Title/Abstract))) AND (("2020"(Date - Publication): "2024"(Date - Publication)))	PubMed	239
Crohn’s Disease AND Gut Microbiome	ScienceDirect	1117
Crohn’s Disease AND Gut Microbiome	MDPI	1195
Crohn’s Disease AND Gut Microbiome	Google Scholar	17700

Selection Process

We transferred all the articles to the Endnote (Clarivate, Philadelphia, Pennsylvania) and removed any duplicates. We screened each article through abstracts and titles. These articles were evaluated for full text and applied inclusion and exclusion criteria. The articles that satisfied all these criteria were shortlisted.

Inclusion and Exclusion Criteria

This research is based on a review of papers published between 2020 and 2024 that involved adult patients only. This review includes human and animal studies, and the literature search is restricted to complete free-text articles available in English. Gray literature (unpublished academic papers and reports), literature with pediatric CD, and papers published in languages other than English are excluded from this review. Moreover, all articles published before 2020 and those limited due to the pagination limit were excluded from the review.

Quality Assessment

The shortlisted articles were checked using the relevant appraisal tools to assess their quality. Observational studies were evaluated using the Newcastle-Ottawa Scale (NOS) tool [22], systematic reviews were assessed using the Assessment of Multiple Systematic Reviews (AMSTAR) [23], and narrative reviews were evaluated by the Scale for the Assessment of Narrative Review (SANRA) [24]. A final list of articles was included in the systematic review only after the quality appraisal was satisfied. Table 2-5 shows a detailed overview of finalized articles and the tools used to assess their quality, and each article was qualified only if the score was above 65%.

**Table 2:** NOS for cross-sectional study NOS: Newcastle-Ottawa Scale; N/A: not available

Criteria	Gao et al. [25]
Selection	-
Representativeness	1
Sample size	1
Ascertainment of exposure	1
Non-respondents	N/A
Comparability	-
Control of confounding	2
Outcome	-
Assessment of outcome	1
Statistical analysis	1
Data completeness	1
Total	8/9 (89%)

**Table 3:** NOS for observational cohort and longitudinal prospective cohort study NOS: Newcastle-Ottawa Scale

Criteria	Buffet et al. [26]	Ma et al. [27]
Selection	-	-
Representativeness of exposed cohort	1	1
Representativeness of non-exposed cohort	0	1
Ascertainment of exposure	1	1
Demonstration that the outcome of interest was not present at the start of the study	1	1
Comparability	-	-
Comparability of cohorts on the basis of the design or analysis	1	1
Comparability of cohort in terms of confounding factors	1	1
Outcome	-	-
Assessment of outcome	1	1
Follow-up long enough for outcomes to occur	1	1
Adequacy of follow-up of cohorts	1	1
Total	8/9 (89%)	9/9 (100%)

**Table 4:** AMSTAR tool for systematic review AMSTAR: Assessment of Multiple Systematic Reviews

AMSTAR Criteria	Hu et al. [28]
A priori design	Yes
Duplicate study selection and data extraction	Yes
Comprehensive literature review	Yes
Unpublished grey reports sought	No
List of included and excluded studies provided	Yes
Characteristics of individual studies provided	Yes
Scientific quality of studies assessed and documented	Yes
Scientific quality of studies used for conclusion	Yes
Statistical method appropriate	Yes
Likelihood of publication bias assessed	Yes
Conflict of interest declared in both systematic review and included studies	Yes
Total score	10/11 (91%)

**Table 5:** SANRA tool for narrative reviews SANRA: Scale for Assessment of Narrative Review Articles

Study	Justification of the Article's Importance for the Readership	Statement of Concrete Aims or Formulation of Questions	Description of the Literature Search	Referencing	Scientific Reasoning	Appropriate Presentation of Data	Total
Nunez et al. [29]	2	1	1	2	2	1	9/12 (75%)
Sugihara et al. [30]	2	2	1	2	1	2	10/12 (83%)
Starz et al. [31]	2	0	1	2	2	2	9/12 (75%)
Zhang et al. [32]	2	1	2	2	2	2	11/12 (92%)
Cheng et al. [33]	2	1	1	2	2	1	9/12 (75%)
He et al. [34]	2	2	2	2	2	2	12/12 (100%)
Yuan et al. [35]	2	1	1	2	2	2	10/12 (83%)

Data Collection

The finalized articles were extracted manually, and quality analysis was done to obtain the output necessary for the review.

Results

Study Identification and Selection

**Figure 1:**
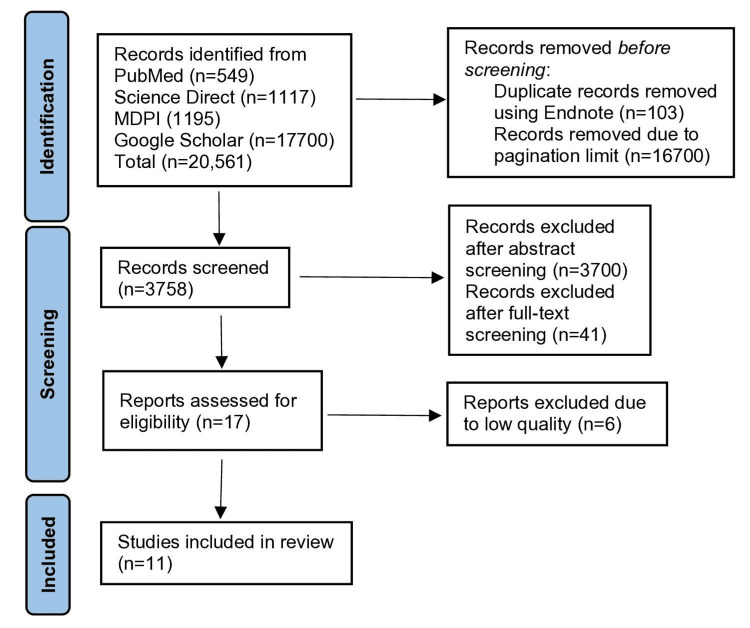
PRISMA flowchart PRISMA: Preferred Reporting Items for Systematic Reviews and Meta-analysis; MDPI: Multidisciplinary Digital Publishing Institute; n: total number

We detected a total number of 20,561 relevant articles across the databases and registers. Among them, we removed 103 duplicates by using Endnote. Also, we removed 16,700 articles from Google Scholar, as we could not access them due to the pagination limit. Then, we screened the remaining articles for titles and abstracts, eliminated 3,700 articles inconsistent with the research topic, and filtered the number of articles to 58. Among them, 41 articles were excluded after full-text screening. Finally, these 17 shortlisted articles were assessed for quality using relevant tools, and we finalized 11 articles for our review. This systematic review was conducted following the guidelines of the PRISMA checklist, which ensures the reliability and transparency of the data we presented. Figure 1 presents the PRISMA flowchart for the current review.

Characteristics of the Study

We have reviewed the final 11 articles, one of which is a cross-sectional study [25], one of which is an observational study [26], one longitudinal prospective cohort study [27], and one systematic review [28]. The other seven are review articles [29-35]. Table 6 summarizes all 11 papers and their characteristics.

**Table 6:** Characteristics of finalized papers CD: Crohn's disease; IBD: inflammatory bowel disease; GM: gut microbiome; miRNA: microribonucleic acid; SCFAs: short-chain fatty acids; PICRUSt: phylogenetic investigation of communities by reconstruction of unobserved states; FMT: fecal microbiota transplantation; UC: ulcerative colitis

Author and Year of Publication	Type of Study	Quality Appraisal Tool Used	Number of Total Patients/Participants	Purpose of the Study	Results and Conclusions
Gao et al. 2023 [25]	Cross-sectional study	The Newcastle-Ottawa Scale (NOS)	30 patients with CD-540 samples from various intestinal layers	Investigate the interactions between host and microbes in CD patients.	Dysbiosis is observed in different tissues and results in the progression of CD.
Buffet-Bataillon et al. 2022 [26]	Prospective observational study	The Newcastle-Ottawa Scale (NOS)	259 CD patients	To assess the stability of microbes and its relation to CD symptom aggravation.	Symptom exacerbation related to reduced SCFAs and increased pro-inflammatory microbes.
Ma et al. (Lu-equal contribution) 2022 [27]	Longitudinal prospective cohort study	The Newcastle-Ottawa Scale (NOS)	40 CD patients and 30 healthy controls	To understand the GM and their role in the pathogenesis of CD.	PICRUSt analysis for various microbial metabolic pathways and their involvement in the perpetuation of CD.
Hu et al. 2022 [28]	Systematic review	Assessment of Multiple Systematic Reviews (AMSTAR)	-	Role of dysbiosis and the metabolites involved in CD/IBD.	The resulting dysbiosis and reduction of beneficial metabolites are critical to the pathogenesis of CD.
Nunez-Sanchez et al. 2022 [29]	Review	Scale for the Assessment of Narrative Review Articles (SANRA)	-	Review the role of GM in the pathogenesis of CD.	The Study confirms that GM significantly leads to a progression of CD, and this idea can be used to uncover treatment options like probiotics for CD.
Sugihara et al. 2021 [30]	Review	Scale for the Assessment of Narrative Review Articles (SANRA)	-	Recent advancements in the role of GM in the pathogenesis of CD.	The review emphasizes the role of GM in the progression of CD and potential treatment using this concept.
Starz et al. 2021 [31]	Review	Scale for the Assessment of Narrative Review Articles (SANRA)	-	Examine the role of GM in CD progression.	This review confirms dysbiosis and its role in the progression of inflammation in CD.
Zhang et al. 2022 [32]	Review	Scale for the Assessment of Narrative Review Articles (SANRA)	-	Outlines the role of GM in CD pathogenesis and other factors involved like genetics and environmental variables.	This review explains the interplay between GM and other factors that significantly influence the onset and progression of CD.
Cheng et al. (Xu contributed equally) 2023 [33]	Review	Scale for the Assessment of Narrative Review Articles (SANRA)	-	Emphasize the role of GM in the immune process of CD.	This review highlights the role of GM and the role of miRNA in the progression of CD.
He et al. 2022 [34]	Review	Scale for the Assessment of Narrative Review Articles (SANRA)	1764	To explore the current knowledge of GM and the influence of dietary patterns on CD pathogenesis.	This review finds the relevant literature on the involvement of various diets on immune processes and how this influences intestinal microbes and leads to a progression of inflammation.
Yuan et al. 2023 [35]	Review	Scale for the Assessment of Narrative Review Articles (SANRA)	-	This Study explores the role of GM and recent advances in applying this information as a therapeutic role in CD.	This article confirms that dysbiosis is a result of inflammation in CD, and this, in turn, leads to a progression of CD. It also highlights the use of probiotics and FMT in the treatment of CD.

Discussion

The CD is a complicated and continuous inflammatory condition with multiple etiologies [4,5]. The interaction between genetics and environmental variables influences the pathogenesis of the disease, which elicits an immune response responsible for the disease progression [29]. Although various treatment options are available for maintaining inflammation, recent research is explicitly working to identify non-pharmacological treatment options, which mainly focus on gut microbiome manipulation [34]. GM plays a significant role in the disease onset and progression by affecting inflammation through various pathways [12,13]. Therefore, current studies are focused on dietary changes and offer prebiotics (supplements that promote the growth of gut bacteria), probiotics (beneficial gut bacteria), and synbiotics (a mixture of both prebiotics and probiotics) as a potential remedy to prevent the progression and prevent complications of CD [35]. This review mainly focuses on how GM is responsible for the progression of inflammation in CD through multiple mechanisms.

Immune Cells in CD

GM plays an essential role in maintaining the integrity of the immune system by activating anti-inflammatory components [29]. However, the dysbiosis caused by CD reduces beneficial bacteria and increases harmful/destructive bacteria, which causes an imbalance in the immune system [11]. This disparity in the GM leads to the aggravation of the immune system, which triggers the production of pro-inflammatory cytokines. These modified cells initiate and perpetuate inflammation in CD [29].

**Figure 2:**
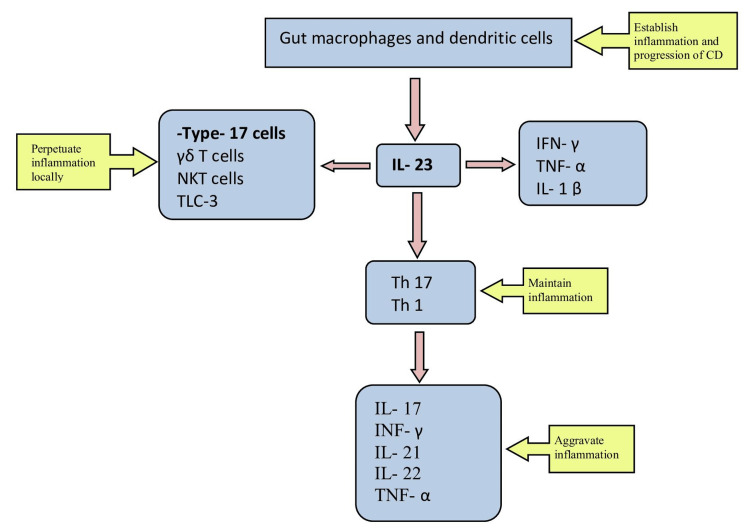
Immune cells in CD Image credits: Sai Pavitra Paidimarri CD: Crohn's disease; IL-1β: interleukin 1 beta cell; γδ T cell: gamma delta T cell; IFN-γ: interferon-gamma cell; TNF-α: tumor necrosis factor-alpha cell; Th: T-helper; NKT: natural killer T-cells; ILC-3: type 3 innate lymphoid cells

The primary cytokines involved in exacerbating CD inflammation are IL-17 and IL-23. Gut macrophages and dendritic cells induce the production of IL-23, TNF-α, and IL-1 beta, which play a role in the progression of CD by intensifying the inflammatory process. IL-23 is involved in chronic inflammation and its deterioration by initiating Th 17, Th 1, and type 17 cells. Th 17, in turn, leads to the activation of IL-17 and other pro-inflammatory cytokines such as IFN-γ, TNF-α, IL-21, and IL-22. The following flowchart (Figure 2) shows the immune process involved in CD [29].

On the other hand, regulatory T (Treg) cells balance gut homeostasis by suppressing the immune cells and acting as anti-inflammatory cells [29].

Gut Barrier Integrity and GM

The preservation of gut barrier integrity is paramount to protect it against the invasion of pathogenic organisms and to maintain optimum immune function in the host [35]. Also, the beneficial GM plays a crucial role in protecting the barrier function by reducing intestinal permeability and producing protective cytokines. In CD, dysbiosis causes the weakening of the intestinal protective barrier, thereby increasing the permeability [28]. For example, CD causes an increase in the *E. coli *population, which causes disintegration of tight junction proteins and apoptosis of gut macrophages. This will eventually amplify the inflammatory response, thereby aggravating the intestinal barrier disintegration [27,34].

Gut Bacteria in CD

Microbes are divided into alpha and beta diversities to understand microbiomes' complex properties and variability. Alpha diversity refers to different kinds of microbes present in a single sample, while beta diversity is the comparison between multiple samples of microbes. In CD patients, there is a reduction in the alpha population, which means there is reduced variability in the microbes, especially *Firmicutes* and *Bacteroidetes*, which are most abundant in the healthy gut [30]. In an observational study [26], 259 patients with CD were divided into G1, G2, and G3 groups, and there was a prominent decrease in alpha diversity as the severity of symptoms increased. This study shows that CD can cause dysbiosis in affected individuals.

A decrease in anti-inflammatory bacteria characterizes dysbiosis in CD, while the pro-inflammatory bacteria are highly increased. Table 7 below shows the data of reduced and increased bacteria and the effects on inflammation by a few bacteria [33].

**Table 7:** Bacteria and their inflammatory effects IL: interleukin; IFN: interferon; Th cells: T-helper cells; Tregs: T-regulatory cells; CD: Crohn's disease

Diminished Bacterial Population	Effects on Inflammation
Faecalibacterium prausnitzii	Produce IL-10 (anti-inflammatory) and decrease IL-12 and IFN (pro- inflammatory)
Blautia faecis	-
Helicobacter pylori	Shift the balance towards anti-inflammatory
Roseburia inulinivorans	Reduce butyrate (increase gut barrier integrity)
Coprococcus comes	-
Lactobacillus reuteri	Induce T-cells, which reduce inflammation
*Clostridium lavalense*	Differentiation and expansion of Tregs (pro-inflammatory)
Enhanced bacterial population	Effects on inflammation
Adhesion invasive *Escherichia coli* (AIEC)	Induce Th 17 (pro-inflammatory)
Segmented filamentous bacteria	Increase production of Th 17 cells
Fusobacteriaceae	Adhere and invade epithelial cells in the colon
Bacteroides fragilis	Induce Th 17 cells
Raminococcus gnavus	Produce capsular polysaccharides- aggravate CD
Clostridium difficile	-
Campylobacter concisus	Release pro-inflammatory cytokines and increase intestinal permeability

Bacterial Metabolites in CD Progression

GM produces various metabolites that are mainly responsible for intestinal epithelial cells' nutritional and immune balance. In dysbiosis caused by CD, the beneficial bacteria are diminished, which decreases these metabolites, thereby affecting the gut cells and leading to the progression of inflammation [28]. The important metabolites involved are short-chain fatty acids, mainly butyrate, acetate, and propionate, bile acids, tryptophan, outer membrane vesicles, microRNA, tight junction proteins, and others. In this review, we will discuss the function of metabolites and their role in CD perpetuation.

Short-Chain Fatty Acids (SCFAs)

These are small molecules produced by GM due to the fermentation of dietary fiber [29]. SCFAs are crucial in molecules that provide energy to the gut cells [31]. They help in maintaining homeostasis in the abdomen through gut barrier integrity, provide energy primarily to colon cells, help in intestinal cell turnover, and play a major in the immunomodulatory process by inducing *Foxp3* expression that is involved in the differentiation of Tregs and produces retinoic acids and TGF-β1 that has anti-inflammatory properties [28]. Among them, the main produced ones are butyrate, acetate, and propionate [30]. According to one cross-sectional study, there was a remarkable reduction in SCFA-producing bacteria such as *Coprococcus, Blautia*, and *Clostridium* and a marked increase in *Proteus* and *E. coli*. This difference was most noted in the advanced patients with CD, and this pattern signifies that dysbiosis is associated with the progression of CD [27].

Butyrate is the primary SCFA produced by GM, which is responsible for gut barrier integrity, differentiation of Tregs, and inhibition of the NFkB pathway, in which inflammatory cells are expressed [32]. The main butyrate-producing bacteria, *Faecalibacterium prausniztii* and *Roseburia hominis*, are significantly reduced by five to 10-fold in the gut of patients with CD [31]. In addition, the pro-inflammatory cytokines, TNF and IFN, reduce the expression of butyrate transporter, monocarboxylate transporter (MCT1), in the inflamed mucosa [30]. Dysbiosis caused by antibiotics used in CD also leads to a reduction of SCFA, especially butyrate, further causing the disintegration of the intestinal barrier and leading to the development of inflammation [35]. In the same way, acetate regulates inflammation and maintains gut immunity [30].

Bile Acids (BAs)

BAs are primarily absorbed in the ileum except for a small portion, metabolized by intestinal microbes [30]. For example, *Firmicutes* produces an enzyme, bile salt hydrolase, which is deficient in CD patients. This leads to a decrease in primary BAs, cholate, and chenoxycholate, worsening the immune response in the gut and increasing inflammation [34]. Bile acid receptor activation induces Treg cells' differentiation and increases IL-10, an anti-inflammatory cytokine. They also reduce IL-1, IL-6, TNF-α, and IFN-γ, the major pro-inflammatory cytokines [29].

Tryptophan

Tryptophan is an essential amino acid precursor for metabolites such as indole, kynurenine (Kyn), and serotonin [28]. Kyn acts on the aryl hydrocarbon receptor (AhR), a critical immune modulator involved in the integrity of the gut barrier [30]. Gut microbes such as *Lactobacillus reuteri *help break tryptophan into indole, which regulates the production of IL-22, which plays a vital role in intestinal stability and inhibits the NFkB pathway. Hence, the dysbiosis caused by CD decreases the tryptophan metabolism, thereby decreasing the expression of AhR and IL-22 and exacerbating inflammation [30].

Outer Membrane Vesicles (OMVs)

OMVs are small vesicles produced by gram-negative bacteria in the intestinal epithelium. The destructive bacteria that increase in CD produce various pro-inflammatory cytokines that lead to the progression of inflammation. For example, *AIEC* secretes OMVs that activate IL-8 cytokines, which facilitate the penetration of the bacteria into the intestinal mucosa. OMVs secreted by *Bacillus fragilis* are responsible for increased pro-inflammatory cytokine IL-8 [29].

MicroRNA (miRNA)

miRNAs are tiny RNAs responsible for modulating the genes, affecting the integrity and inflammation of the intestinal barrier. Various miRNAs express differently in different tissues and have different functions. For example, miR-155 hinders the proliferation of Th 17 and Th 1 cells, whereas miR-21 increases the production of TNF alpha, that are pro-inflammatory and aggravates the inflammation in intestinal cells. The miRNAs are altered in patients with CD; hence, they improve or worsen the progression of inflammation in CD [33].

Tight Junction Proteins (TJPs)

TJPs enhance the interconnection between intestinal cells and maintain the integrity of the gut barrier. Few TJPs, such as claudin, occludin, and ZO-1, are significantly reduced in CD patients, which weakens the gut lining and exacerbates inflammation [28]. Also, gut bacteria such as *L. reuteri *protect the TJPs in the intestinal mucosa; thus, dysbiosis increases inflammation [28].

Some numerous other metabolites and receptors are altered in CD patients and are responsible for the perpetuation of inflammation. Various research trials are being done to understand such mechanisms, which will help uncover different treatment options for CD. Recently, it has been identified that Vitamin D receptors (35) and mucus-associated proteins such as carcinoembryonic antigen-related cell adhesion molecule1 (CECAM1) play a vital role in the pathogenesis of CD [25]. The sulfur-reducing bacteria that produce hydrogen sulfide as a metabolite, a pro-inflammatory mediator, increased in CD patients [32]. Also, high oxygen and nutritional changes in inflammatory tissue are involved in disease onset and progression [30].

Gut Fungi, Virome, and Helminths

While much attention was given to bacteria all these years, emerging evidence indicates the involvement of other organisms that harbor in the GM, including various fungi, viruses, and parasites, in the initiation and progression of CD. For instance, gut mycobiome dysbiosis produces abundant fungi such as *Candida* and *Ascomycetes*. The hyphae and other proteins, such as candidolysin of Candida, are involved in the propagation of inflammation [29]. In addition, there is an increased population of various pathogenic phages in the gut due to inflammation, which might be a cause of CD. Also, several studies suggest that the defense mechanisms of helminths are protective for the onset of CD. Finally, the interplay between all these organisms might exacerbate inflammation in CD [33].

Limitations

The main limitation of this review is that no papers with clinical trials were involved in the data extraction. Another limitation is that we could access only 1000 articles among 17,700 articles found in Google Scholar, which could result in the loss of some relevant studies. Other organisms of GM, including fungi, viruses, and helminths, are involved, but their involvement still needs to be fully established. Finally, this study involves papers published on platforms with free text and in the English language, which can limit the scope of the search and literature.

## Conclusions

This review focuses on how the intestinal microbial imbalance leads to the progression of inflammation observed in CD. The articles we reviewed revealed that dysbiosis affects the immune processes of the gut by reducing the anti-inflammatory cytokines and increasing the pro-inflammatory cytokines, leading to the prolongation of inflammation. Due to a decrease in beneficial bacteria such as *Faecalibacterium* and *Roseburia*, there is a decline in specific metabolites such as SCFAs, bile acids, tryptophan, and others that are involved in the maintenance of both immune activity and the barrier integrity of the intestine. Furthermore, CD leads to an increase in harmful bacteria such as AIEC, which are involved in the induction and propagation of inflammatory cytokines that trigger the perpetuation of the inflammatory process in CD. In addition, several dietary patterns, antibiotics, and genetic factors affect the GM and are associated with the severity of CD. Understanding the contribution of GM in CD is essential, as it can help us develop new treatment strategies that could potentially induce long-term remission in patients. Numerous studies are being conducted on the gut microbiome, their metabolites, dysbiosis, and mechanisms in CD, which can provide a further understanding of the disease process that can aid in treating CD.

This study provides insight into the existing knowledge of how the gut microbiome affects CD. It can also serve as a significant resource for an overview of what is already known about the disease and as a future reference for conducting further research studies on the pathogenesis and management of CD.
